# Supplementary material for: FERN – a Java framework for stochastic simulation and evaluation of reaction networks
Source: BMC Bioinformatics. 2008 Aug 29;9:356. doi: 10.1186/1471-2105-9-356 (PMC2553347; doi:10.1186/1471-2105-9-356)
Supplement: Additional file 1 — FERN distribution, Version 1.3. This archive contains the FERN source code and binaries as well as documentation and example models in FernML and SBML. [file 1471-2105-9-356-S1.zip › fern/doc/javadoc/index-files/index-18.html]

T-Index


---


|  |  |  |
| --- | --- | --- |
| **Overview** \| Package \| Class \| Use \| **Tree** \| **Deprecated** \| **Index** \| **Help** | |  |
| **PREV LETTER**   **NEXT LETTER** | **FRAMES**    **NO FRAMES**     **All Classes** |  |


A B C D E F G H I J L M N O P R S T U V 

---


## **T**

**t** - Variable in class fern.simulation.Simulator: Contains the actual time of the simulation. **TauLeapingAbsoluteBoundSimulator** - Class in fern.simulation.algorithm: There are some possibilities to bind the expected change of the propensities by a value epsilon in order to fulfill the leap condition. **TauLeapingAbsoluteBoundSimulator(Network)** - Constructor for class fern.simulation.algorithm.TauLeapingAbsoluteBoundSimulator: **TauLeapingRelativeBoundSimulator** - Class in fern.simulation.algorithm: There are some possibilities to bind the expected change of the propensities by a value epsilon in order to fulfill the leap condition. **TauLeapingRelativeBoundSimulator(Network)** - Constructor for class fern.simulation.algorithm.TauLeapingRelativeBoundSimulator: **TauLeapingSpeciesPopulationBoundSimulator** - Class in fern.simulation.algorithm: There are some possibilities to bind the expected change of the propensities by a value epsilon in order to fulfill the leap condition. **TauLeapingSpeciesPopulationBoundSimulator(Network)** - Constructor for class fern.simulation.algorithm.TauLeapingSpeciesPopulationBoundSimulator: **theta(double)** - Method in class fern.cytoscape.CytoscapeColorChangeObserver: **theta(double)** - Method in class fern.example.CellGrowthObserver: **theta(double)** - Method in class fern.network.sbml.SBMLEventHandlerObserver: **theta(double)** - Method in class fern.simulation.observer.AmountAtMomentObserver: Records the amounts of the species and puts it into the histogram. **theta(double)** - Method in class fern.simulation.observer.FireTypeObserver: Do nothing. **theta(double)** - Method in class fern.simulation.observer.InstantOutputObserver: Prints event. **theta(double)** - Method in class fern.simulation.observer.IntervalObserver: If the `theta` method is used, the data is recorded here. **theta(double)** - Method in class fern.simulation.observer.LeapObserver: Do nothing. **theta(double)** - Method in class fern.simulation.observer.Observer: Gets called by simulators when a certain moment in time is reached. **theta(double)** - Method in class fern.simulation.observer.RandomNumberGeneratorCallObserver: Do nothing. **thetaEvent()** - Method in class fern.simulation.Simulator: Gets called when the simulator reaches the special time `theta`. **toArray(Collection<Integer>)** - Static method in class fern.tools.NumberTools: Copies the values of the given collection and unboxes them to an array. **toArray(Collection<Double>)** - Static method in class fern.tools.NumberTools: Copies the values of the given collection and unboxes them to an array. **toGnuplot(String[], String[])** - Method in class fern.benchmark.Benchmark: Adds the benchmark data without conversion to a new `GnuPlot` object. **toGnuplot(GnuPlot, String[], String[])** - Method in class fern.benchmark.Benchmark: Adds the benchmark data without conversion to a given `GnuPlot` object. **toGnuplot()** - Method in class fern.simulation.observer.AmountAtMomentObserver: **toGnuplot(GnuPlot)** - Method in class fern.simulation.observer.AmountAtMomentObserver: **toGnuplot()** - Method in class fern.simulation.observer.FireTypeObserver: **toGnuplot(GnuPlot)** - Method in class fern.simulation.observer.FireTypeObserver: **toGnuplot()** - Method in interface fern.simulation.observer.GnuPlotObserver: Creates a new `GnuPlot` object and passes the actual observer data to it. **toGnuplot(GnuPlot)** - Method in interface fern.simulation.observer.GnuPlotObserver: Passes the actual observer data to a `GnuPlot` object. **toGnuplot()** - Method in class fern.simulation.observer.IntervalObserver: **toGnuplot(GnuPlot)** - Method in class fern.simulation.observer.IntervalObserver: **toGnuplot()** - Method in class fern.simulation.observer.LeapObserver: **toGnuplot(GnuPlot)** - Method in class fern.simulation.observer.LeapObserver: **toGnuplot()** - Method in class fern.simulation.observer.RandomNumberGeneratorCallObserver: **toGnuplot(GnuPlot)** - Method in class fern.simulation.observer.RandomNumberGeneratorCallObserver: **toGnuPlotAsHistogram(String[], String[])** - Method in class fern.benchmark.Benchmark: Adds the benchmark data as histogram to a new `GnuPlot` object. **toGnuPlotAsHistogram(GnuPlot, String[], String[])** - Method in class fern.benchmark.Benchmark: Adds the benchmark data as histogram to a given `GnuPlot` object. **toGnuplotRecent()** - Method in class fern.simulation.observer.IntervalObserver: Creates a new `GnuPlot` object and passes the recent observer data to it. **toGnuplotRecent(GnuPlot)** - Method in class fern.simulation.observer.IntervalObserver: Passes the recent observer data to a `GnuPlot` object. **toString()** - Method in class fern.analysis.ShortestPath.Path: **toString()** - Method in class fern.network.sbml.MathTree.ConstLeaf: **toString()** - Method in class fern.network.sbml.MathTree.GlobalLeaf: **toString()** - Method in class fern.network.sbml.MathTree.InnerNode: **toString()** - Method in class fern.network.sbml.MathTree.VarLeaf: **toString()** - Method in class fern.network.sbml.SBMLEventHandlerObserver: **toString()** - Method in class fern.simulation.algorithm.IndexedPriorityQueue: **toString()** - Method in class fern.simulation.observer.AmountAtMomentObserver: **toString()** - Method in class fern.simulation.observer.FireTypeObserver: **toString()** - Method in class fern.simulation.observer.IntervalObserver: **toString()** - Method in class fern.simulation.observer.LeapObserver: **toString()** - Method in class fern.simulation.observer.RandomNumberGeneratorCallObserver: **TransposedArrayMatrixAxes** - Class in fern.tools.gnuplot: Implementation of `Axes` to use with transposed array matrixes of any number type (the type of the arrays is inferred by using the reflection api). **TransposedArrayMatrixAxes(Object)** - Constructor for class fern.tools.gnuplot.TransposedArrayMatrixAxes: Create a new `Axes` object containing the given array matrix without labels / styles. **TransposedArrayMatrixAxes(Object, String[], String[])** - Constructor for class fern.tools.gnuplot.TransposedArrayMatrixAxes: Create a new `Axes` object containing the given array matrix with the given labels /styles (either can be `null`). **trigger()** - Method in class fern.network.sbml.SBMLEventHandlerObserver: **trigger()** - Method in class fern.simulation.observer.TriggerObserver: Is executed, whenever a event can trigger (before step and during a theta event). **TriggerObserver** - Class in fern.simulation.observer: **TriggerObserver(Simulator)** - Constructor for class fern.simulation.observer.TriggerObserver

---


|  |  |  |
| --- | --- | --- |
| **Overview** \| Package \| Class \| Use \| **Tree** \| **Deprecated** \| **Index** \| **Help** | |  |
| **PREV LETTER**   **NEXT LETTER** | **FRAMES**    **NO FRAMES**     **All Classes** |  |


A B C D E F G H I J L M N O P R S T U V 

---
